# Adjuvant medical treatment for breast cancer in elderly and old women

**DOI:** 10.1007/s12254-016-0258-4

**Published:** 2016-03-08

**Authors:** Theresa Westphal, Gabriel Rinnerthaler, Brigitte Mlineritsch

**Affiliations:** 3rd medical department, Paracelsus Medical University, Müllner Hauptstraße 48, 5020 Salzburg, Austria

**Keywords:** Breast cancer, Adjuvant, Elderly, Older women

## Abstract

The probability of developing breast cancer increases with age. Therefore, more than 50 % of women with breast cancer are older than 65 years at the time of diagnosis. However, elderly patients are often undertreated and clinical trials for elderly patients in the adjuvant setting are lacking. Elderly patients who are otherwise fit should receive the standard treatment regimen independent of age.

Endocrine therapy should not be withheld from patients by age alone. Thus, there are more adverse events in the elderly population.

The decision on adjuvant chemotherapy should be made taking into consideration the patient’s comorbidities and frailty. A less toxic single-agent regimen may influence overall survival, but are associated with much less toxicity. Trastuzumab has a similar effect in elderly patients to that in younger patients. The risk of cardiotoxicity should be carefully considered in each patient.

## Introduction

Breast cancer is a common malignancy, affecting women of all ages. However, more than 50 % of women with breast cancer are over 65 years of age and around 25 % are over 75 years of age at the time of diagnosis [1, 2]. The probability of dying of breast cancer declines with the age at diagnosis, but the overall mortality increases owing to secondary diagnoses [3].

Although elderly women with breast cancer represent a large group of cancer patients, data suggest that more than 51 % of elderly women are undertreated [4]. In a large cohort study of patients aged 65 or over, 19 % had no triple assessment and 44 % did not undergo oestrogen receptor testing. Patients are less likely to undergo axillary surgery, less likely to have radiotherapy, and more likely to have mono-hormonal therapy [4, 5].

## Biomarkers 

Differences in breast cancer treatment may somehow be a result of different cancer presentations, biology, and hormone expression in the elderly. However, looking at the characteristics of biological cancer more closely, recent data suggest that there might be only small differences in stage, size, axillary node positivity, oestrogen receptor positivity, progesterone receptor status, HER2 negativity, and S‑phase reaction between the age groups [2, 6, 7]. Furthermore, older patients are at a lower risk of local recurrence than younger patients [8].

## General aspects in the treatment of elderly patients with breast cancer

It is important to state that decisions regarding therapy should never be based on age alone. Elderly patients who are otherwise fit should receive the standard treatment regimen independent of age [9]. In any case, multiple factors should be considered to find the right treatment. Not only comorbidities, medication, organ function, nutrition, but physical status and social support also influence the decision. A comprehensive geriatric assessment may be useful in evaluating individual performance [10].

Another problem regarding the optimal treatment in elderly patients is the lack of detailed knowledge. Because older patients usually have more comorbidities, they fail the eligibility criteria and are underrepresented in clinical trials [11]. During the past few years there has been a step toward carrying out more clinical trials for elderly patients. However, no clear guidelines are yet available for the treatment of breast cancer in elderly women.

In the following section we summarize the current options and recommendations for adjuvant medical treatment.

## Endocrine therapy

In 2008 a report tried to explore the potential differences in efficacy, treatment completion, and adverse events in elderly women receiving adjuvant tamoxifen or letrozole for 5 years in the Breast International Group (BIG) 1‑98 trial. Subpopulation Treatment Effect Pattern Plot (STEPP) analysis was used for nearly 5,000 patients to examine the differences in disease-free survival and adverse events according to age. Efficacy results were similar to the overall trial results. Compared with tamoxifen, letrozole significantly improved disease-free survival. Elderly patients were less likely to complete trial treatment. In elderly patients, letrozole had a significantly higher incidence of any grade 3–5 protocol-specified non-fracture adverse events compared with tamoxifen (*p* = 0.002). There were no significant differences in cardiac and thromboembolic events [12].

An increase in bone loss is a well-known problem during treatment with aromatase inhibitors. This is aggravated by the decline in bone mineral density with age. Skeletal status should be assessed when considering aromatase inhibitor therapy. Bisphosphonates, an adequate intake of calcium and vitamin D and a healthy lifestyle are approved options for preventing bone loss. These recommendations apply to patients with a t-score < −2, but also to patients over 75 with one or more additional risk factor for a bone fracture [13].

## Adjuvant chemotherapy

In addition to the many women with oestrogen-positive breast cancer, there is also a high proportion of women who have adverse prognostic markers such as negative estrogen and progesterone status and who are eligible for chemotherapy [14].

As mentioned above, clinical trials that assess the toxicity and efficacy of chemotherapeutic agents in elderly patients are lacking. We have summarised a few trials that might help us to find appropriate chemotherapy regimens in the future.

Standard adjuvant chemotherapy with four cycles of doxorubicin and cyclophosphamide or six cycles of cyclophosphamide, methotrexate, and 5‑fluorouracil have been shown to improve survival from breast cancer in older women. However, these regimen show high toxicity in older patients [15].

Muss et al. [16] compared capecitabine alone with standard chemotherapy (either cyclophosphamide, methotrexate, and fluorouracil or cyclophosphamide or doxorubicin) in women with breast cancer stages I to IIIB. The results showed that patients who took capecitabine were twice as likely to have a relapse as those under the chemotherapy regimen (*p* = 0.02). The overall survival was 86 % vs 91 % in the chemotherapy group. However, twice as many patients receiving standard chemotherapy had moderate-to severe toxicity. The authors concluded that standard adjuvant chemotherapy is superior to capecitabine in elderly patients with early-stage breast cancer.

The CALGB 40101 study compared doxorubicin and cyclophosphamide versus single-agent paclitaxel. The absolute advantage of the standard regimen was 3 % for recurrent free survival and 1 % for overall survival. Despite that, the authors concluded that this trial did not show non-inferiority of paclitaxel [17]. Nevertheless, paclitaxel was much less toxic than the standard regimen with only minimal disadvantage in survival rates.

To reduce cardiac toxicity Freyer et al. [18] described tolerance of adjuvant docetaxel/cyclophosphamide in a retrospective observational study. The authors suggest that docetaxel/cyclophosphamide might be feasible and safe for selected patients over 70 years of age with only few cases of neutropenic fever during the period of observation.

It is possible that many patients would profit from agents that are nearly as effective as the standard regimen, but have much lower toxicity.

Until now, decision-making has depended mainly on the individual’s physical constitution, frailty, and secondary diagnoses. Further trials on less toxic agents are urgently needed.

## Trastuzumab

The probability of HER2-positive tumors in elderly patients seems to be similar to that in younger ones [7]. Patients over 60 years of age seem to profit from a treatment with trastuzumab as much as the whole study population [19]. A low-toxicity regimen that might be appropriate for older patients was examined in a large phase 2 study. It contains weekly paclitaxel for 12 weeks with trastuzumab and showed good tumor control with a 3-year recurrence-free interval of 99 % [20]. One side effect of trastuzumab is of particular interest in older patients. Trastuzumab is known to induce cardiotoxicity, which leads to a decreased left ventricular ejection function and may induce heart failure. However, it does not seem to occur more frequently in older patients than in a younger population and seems to be reversible in most cases [21]. Nevertheless, conclusive data on cardiotoxicity in older patients are lacking.

To decrease the risk of an adverse cardiac event, ESMO guidelines favor the use of trastuzumab in combination with non-anthracycline-based regimen [22].

## Conclusion

In general, treatment decisions for elderly patients should never be limited to age alone, but should include physical constitution, comorbidities, frailty, social assessment, and geriatric assessment. Endocrine therapy should not be withheld from patients by age alone. Thus, there are more adverse events in the elderly population. The decision on adjuvant chemotherapy should be made taking into consideration the patient’s comorbidities and frailty. A less toxic single-agent regimen may influence overall survival, but is associated with much lower toxicity. Trastuzumab has a similar effect in elderly patients to that in younger ones. The risk of cardiotoxicity should be carefully considered in each patient. In general, guidelines for the treatment of breast cancer in the elderly are lacking and more clinical trials are needed.
